# Fully automated template matching method for ECG-free heartbeat detection in cardiomechanical signals of healthy and pathological subjects

**DOI:** 10.1007/s13246-025-01531-3

**Published:** 2025-03-13

**Authors:** Salvatore Parlato, Jessica Centracchio, Daniele Esposito, Paolo Bifulco, Emilio Andreozzi

**Affiliations:** 1 Department of Electrical Engineering and Information Technologies, University of Naples Federico II, Via Claudio, 21, I-80125 Naples, Italy; 2 Department of Information and Electrical Engineering and Applied Mathematics, University of Salerno, Via Giovanni Paolo II, 132, I-84084 Fisciano, Italy

**Keywords:** Cardiac monitoring, Heart rate, Gyrocardiography, Seismocardiography, Forcecardiography, Template matching

## Abstract

Cardiomechanical monitoring techniques record cardiac vibrations on the chest via lightweight electrodeless sensors that allow long-term patient monitoring. Heartbeat detection in cardiomechanical signals is generally achieved by leveraging a simultaneous electrocardiography (ECG) signal to provide a reliable heartbeats localization, which however strongly limits long-term monitoring. A heartbeats localization method based on template matching has demonstrated very high performance in several cardiomechanical signals, with no need for a concurrent ECG recording. However, the reproducibility of that method was limited by the need for manual selection of a heartbeat template from the cardiomechanical signal by a skilled operator. To overcome that limitation, this study presents a fully automated version of the template matching method for ECG-free heartbeat detection, powered by a novel automatic template selection algorithm. The novel method was validated on 256 Seismocardiography (SCG), Gyrocardiography (GCG), and Forcecardiography (FCG) signals, from 150 healthy and pathological subjects. Comparison with all existing methods for ECG-free heartbeat detection was carried out. The method scored sensitivity and positive predictive value (PPV) of 97.8% and 98.6% for SCG, 96.3% and 94.5% for GCG, 99.2% and 99.3% for FCG, on healthy subjects, and of 85% and 95% for both SCG and GCG on pathological subjects. Statistical analyses on inter-beat intervals reported almost unit slopes (R^2^ > 0.998) and limits of agreement within ± 6 ms for healthy subjects and ± 13 ms for pathological subjects. The proposed automated method surpasses all previous ECG-free approaches in heartbeat localization accuracy and was validated on the largest cohort of pathological subjects and the highest number of heartbeats. The method proposed in this study represents the current state of the art for ECG-free monitoring of cardiac activity via cardiomechanical signals, ensuring accurate, reproducible, operator-independent heartbeats localization. MATLAB^®^ code is released as an off-the-shelf tool to support a more widespread and practical use of cardiomechanical monitoring in both clinical and non-clinical settings.

**Supplementary Information:**

The online version contains supplementary material available at 10.1007/s13246-025-01531-3.

## Introduction

Cardiac monitoring is a critical aspect of healthcare, providing essential information on heart function and related pathologies [1]. Continuous, long-term cardiac monitoring has long been recognized as a critical task to improve the diagnosis and treatment of cardiac diseases [2–4]. The gold standard technique for heart rate monitoring in clinical routine is the electrocardiography (ECG), which records the electrical activity of the heart via electrodes placed on body surface [5]. Heart rate estimation is based on heartbeat detection, which consists of locating R-peaks in ECG signals [4, 6, 7]. For this purpose, the most popular approach is the well-known Pan and Tompkins algorithm [4, 8]. However, ECG has several limitations for continuous long-term monitoring, such as: patients discomfort due to the use of electrodes and wires; loss of stable electrical contact with subjects’ skin due to electrodes detachment, subjects’ movements, drying of conductive gel; susceptibility to electromagnetic interferences. These limitations make ECG unattractive for continuous, long-term cardiac monitoring, particularly in non-clinical settings [6–11]. For this reason, alternative methods for cardiac monitoring have been developed. An alternative to ECG for cardiac monitoring is photoplethysmography (PPG). PPG is a noninvasive optical technique that measures changes in blood volume in the microvascular bed of tissues [12]. The operating principle of PPG involves using a light-emitting diode (LED) to illuminate the skin, usually on the fingertip or earlobe, and a photodetector to measure the amount of light transmitted or reflected. Changes in light absorption correspond to pulsatile changes in blood volume with each heartbeat. Generally, heart rate measurement via PPG involves time localization of specific markers on the PPG signal waveform, such as the systolic peak, which are related to important cardiac cycle events. Heart rate monitors based on PPG are available as alternatives to ECG, as well as in the form of wearable devices (e.g., smartwatches and fitbands). However, their strong sensitivity to motion artifacts usually limits their application in non-clinical settings. In addition, their high power consumption makes them unsuitable for continuous long-term monitoring, especially in wearable devices, where PPG is rather used in an intermittent way to preserve battery life. Moreover, external factors, such as ambient light and skin tone, can affect the quality of PPG signals, posing problems for consistent and reliable heart rate monitoring [12–18].

Further alternatives to ECG for the non-invasive assessment of cardiac function are the cardiomechanical monitoring techniques [19]. These techniques monitor the mechanical activity of the heart by recording the tiny vibrations induced onto the chest wall by the beating heart. Analysis of these cardiac vibrations allows the detection of specific events in the cardiac cycle, such as the opening and closure of heart valves. Cardiomechanical monitoring comprises different techniques. Phonocardiography (PCG) records cardiac vibrations in the acoustic range, i.e. the heart sounds, by means of electronic stethoscopes, and has been shown to be a valuable tool for both ambulatory and remote monitoring [20–24]. Ballistocardiography (BCG) records the recoil forces of the body in reaction to cardiac ejection of blood into the vasculature, capturing the whole-body vibrations due to small displacements of the body center of mass [25–27]. Seismocardiography (SCG) measures the small accelerations of the chest caused by the heartbeats via high-precision accelerometers placed onto subjects’ chests [28–35]. Modern accelerometers, now more sensitive and miniaturized, can be integrated into wearable devices, making SCG a practical technique for continuous cardiac monitoring [36]. Similarly to SCG, Gyrocardiography uses small, lightweight gyroscopes to detect the rotational movements of the chest due to heartbeats, providing additional information on the three-dimensional dynamics of heart motion [37, 38]. Triaxial accelerometers and gyroscopes are usually available in single chips known as inertial measurement units (IMU), which allow novel techniques to monitor simultaneously both linear and rotational movements of the chest induced by cardiac activity. Kinocardiography (KCG) is one such technique: it uses two IMUs, one placed onto the chest and the other onto the abdomen, to obtain 12-axis measurements of body vibrations caused by cardiovascular activities [39, 40]. Recently, a novel technique has been proposed, which was named Forcecardiography (FCG) as it uses specifically designed force sensors to capture the tiny forces induced on the chest wall by cardiac and respiratory activities. FCG is able to sense forces in a broad frequency range, which allows it to monitor respiration, infrasonic cardiac vibrations, and heart sounds, all simultaneously from a single site onto the chest [41–45].

The measurement of instantaneous heart rate is based on heartbeats detection. In ECG signals this task is usually performed by identifying R peaks. In cardiomechanical monitoring techniques, heartbeats detection is usually performed via beat-by-beat identification of specific events of the cardiac cycle, such as the opening and closure of heart valves, which correspond to specific peaks and valleys in cardiomechanical signals. Localizing these events makes it possible not only to calculate the instantaneous heart rate, but also to assess other cardiac time intervals (CTI) of clinical relevance e.g., pre-ejection period, isovolumic contraction time, left ventricular ejection time, total systolic time, isovolumic relaxation time [32, 36, 42, 46, 47]. Nonetheless, several factors can limit markers identification in cardiomechanical signals. Indeed, these signals very often exhibit low signal-to-noise ratios (SNR), as well as alterations in waveform morphology due to pathologies, changes in posture and sensors placement, cardiac response to respiratory activity (cardio-respiratory interactions) [48–52]. All these disturbing factors make the heartbeat detection and instantaneous heart rate monitoring tasks fairly complex. To address these issues, it is common practice in cardiomechanical monitoring to acquire a concurrent ECG lead in order to provide a reliable time reference (i.e., R peaks) for accurate heartbeat localization and subsequent annotation of specific cardiac cycle events [37], [53–55]. However, the need for a simultaneous ECG recording jeopardizes the most attractive benefits of cardiomechanical monitoring techniques, such as their electrodeless operation, which enables continuous, long-term cardiac monitoring by overcoming the typical limitations of ECG.

Few methods for heartbeat detection have been proposed in the literature that do not rely on a reference ECG recording. These include approaches based on signal envelope extraction via wavelet transforms [56], Hilbert and Fourier transforms [57–59], clustering [60], autoregressive models [61], autocorrelated differential algorithm [46], variational mode decomposition [62, 63], matched filtering [64], hidden Markov models [65], template-based detection [66], maximum a posteriori detection [67], rule-based detection [68], as well as machine learning and deep learning methods [69–71]. The main limitations of these methods lie in their high computational complexities, especially those based on artificial intelligence, and in their limited performance assessment. Indeed, they have only been tested on relatively small cohorts of healthy subjects, hence neither extensive assessment on larger cohorts, nor testing on pathological subjects has been carried out for these methods, which limits the reliability of these methods.

Recently, some of the authors presented a novel ECG-free heartbeat detection method based on a template matching approach [72–75]. The method localizes heartbeats in a cardiomechanical signal by searching for matches with an appropriately selected heartbeat template. Specifically, normalized cross-correlation (NCC) is used as a measure of local similarity of the signal with the heartbeat template, and local maxima are detected in NCC to localize the heartbeats, and eventually measure the inter-beat intervals. This simple yet powerful method has the interesting advantage of requiring no a priori knowledge of signal morphology, by obviating the need for identifying specific markers of heartbeats that may not clearly appear in cardiomechanical signals, especially in pathological subjects [48]. This feature gives the method a fair degree of robustness to inter-subject variability and allows it to be applied on different cardiomechanical signals, such as SCG, GCG, and FCG. This ECG-free method has demonstrated very high performance on a large cohort of pathological subjects, both for measuring inter-beat intervals (IBIs) [72, 73, 75] and for estimating heart rate variability indices [74]. Its main limitation lies in the need for manual selection of a heartbeat template, which may limit the overall objectivity and reproducibility of the method if the users find difficulties in adhering to the guidelines provided for the manual template selection. Table 1 compares the cohorts enrolled in the studies, in terms of number of healthy and pathological subjects. It is worth noting that only two studies have been carried out on cohorts of pathological subjects [59, 60], which however had a very small sample size compared to the present study. Concerning the healthy subjects, the majority of the studies also had limited sample sizes.

**Table 1:** Comparison of studies on ECG-free heartbeat detection in SCG and GCG signals: methods and cohort size

Study	Method	Cohort size	
		Healthy subjects	Pathological subjects
Jia et al., 2015 [61]	Autoregressive models	10	0
Tadi et al., 2016 [57]	Hilbert Transform	30	0
Wahlstrom et al., 2017 [65]	Hidden Markov Models	66	0
Hurnanen et al., 2018 [56]	Wavelet Envelope + Dynamic Balancing	66	0
Tadi et al., 2019 [59]	Hilbert Transform + ICA	0	19
Kaisti et al., 2019 [60]	Clustering	29	12
D’Mello et al., 2019 [46]	Autocorrelated differential algorithm	25	0
Cocconcelli et al., 2020 [66]	Template-based detection	20 + 13	0
Thakkar & Sahoo, 2020 [69]	Naïve Bayes/Support Vector Machine/Logistic Regression	20	0
Chen et al., 2021 [70]	Deep learning BiLSTM Network	20	0
Choudary et al., 2021 [62]	Variational mode decomposition	20	0
Scarpetta et al., 2022 [64]	Matched filtering	8	0
Duraj et al., 2022 [71]	ResNet-based convolutional neural network	20	0
Milena et al. 2023 [58]	Hilbert Transform	21	0
Centracchio et al., 2023 [72]	Manual template-matching	0	95
Parlato et al., 2023 [73]	Manual template-matching	0	95
Centracchio et al., 2024 [75]	Manual template-matching	6	0
Zheng et al., 2024 [63]	Variational mode decomposition	20	0
Schipper et al., 2024[67]	Maximum a posteriori detection	147	0
Pickert et al., 2024 [68]	Rule-based detection	12	0
**Current study**	Automated template-matching	49	95

This study addressed and overcame the limitation of the ECG-free heartbeat detection algorithm described in [72–75], by presenting a fully automated version of the template matching method and assessing its performance on a larger cohort of subjects. A novel automatic template selection algorithm was specifically designed to overcome the limitation of manual template selection, thus ensuring reliable and reproducible results without requiring the intervention of a skilled operator. The novel automated method was validated on a large cohort of both healthy (49) and pathological subjects (95), comprising three public datasets of SCG and GCG signals, and a proprietary dataset of Forcecardiography signals, for a total of 256 cardiomechanical signals, of which 172 had been acquired from patients with one or more valvular heart diseases (VHDs). The performances of the proposed method were assessed via statistical analyses, by considering ECG data as the ground truth. Sensitivity and positive predictive value (PPV) were evaluated to assess heartbeat detection performance, while linear regression, correlation, and Bland-Altman analyses of inter-beat intervals were carried out to assess the heartbeat localization accuracy. A MATLAB^®^ code implementation of the full method is available in a public repository [76] to promote its use as an off-the-shelf tool for cardiomechanical signals analysis.

In summary, the main contributions of this study are the design of an automatic template selection algorithm that overcomes the need for manual template selection, and the validation of the fully automated template matching method on the largest cohort of mixed healthy and pathological subjects in the literature.

## Materials and methods

### Datasets

Three public datasets of SCG and GCG signals and a proprietary dataset of FCG signals were considered in this study. For clarity, the datasets were given identification numbers. Dataset #1 [77] comprises ECG, SCG, and GCG signals collected from 29 healthy male subjects (mean age 29 ± 5 years). SCG signals have been measured via a triaxial capacitive digital accelerometer (MMA8451Q, Freescale Semiconductor, Austin, TX, USA), GCG signals via a triaxial gyroscope (MAX21000, Maxim Integrated, San Jose, CA, USA), and ECG signal via an ECG front-end (ADS1293, Texas Instruments, Dallas, TX, USA). All signals have been acquired simultaneously at a sampling frequency of 800 Hz, from subjects at rest in supine position, with sensors attached to the sternum via double-sided tape.

Dataset #2 [78] consists of prolonged recordings of dorso-ventral SCG, ECG, and respiratory signals from 20 healthy subjects (12 males, 8 females aged between 19 and 30 years) at rest in supine position. SCG signals have been measured via a triaxial accelerometer (LIS344ALH, STMicroelectronics, Geneva, Switzerland), and respiratory signals via a thoracic piezoresistive band (SS5LB sensor, BIOPAC Systems Inc, Santa Barbara, CA, USA). All signals have been sampled simultaneously at 5 kHz via a Biopac MP36 system (BIOPAC Systems Inc, Santa Barbara, CA, USA). Each subject’s recording is approximately 1 h long and comprises 3 segments: a 5-minute baseline segment acquired from the subject at rest (segments IDs: b001-b020), a 50-minute segment acquired from the subject while listening to classical music (segments IDs: m001-m020), and a further 5-minute segment acquired from the subject at rest after listening to music (segments IDs: p001-p020). In this study, only the 50-minute segments (segments IDs: m001-m020) were analyzed.

Dataset #3 [79] includes ECG, SCG, and GCG records of 100 patients (59 males, 41 females, age 68 ± 14 years) with VHDs (aortic valve stenosis, aortic valve regurgitation, mitral valve stenosis, mitral valve regurgitation, and tricuspid valve regurgitation). Data have been collected at medical centers in China and USA, from subjects at rest in supine position during quiet breathing. SCG, GCG, and ECG recordings have been simultaneously acquired via a Shimmer 3 ECG module (Shimmer Sensing, Dublin, Ireland) attached on subjects’ chest, which comprised an IMU and a biopotential circuit. The signals have been sampled at 256 Hz (patients #CP-01 to #CP-70 and #UP-01 to #UP-21) and at 512 Hz (patients #UP-22 to #UP-30). As in [72, 73] some recordings were excluded from the analysis due to extremely poor signal quality.

Dataset #4 comprises FCG and ECG signals acquired in a previous study [41]. FCG signals have been collected from six healthy individuals, during quiet breathing while comfortably seating on a chair, by using an FCG sensor placed on their chest around the fifth intercostal space on the midclavicular line, secured via medical adhesive tape and an elastic band. ECG lead II signals have been measured using a multi-parameter patient monitor (Propaq Encore^®^, Welch Allyn^®^, New York, NY, USA). Data have been acquired simultaneously using a National Instruments NI-USB4431 DAQ board (National Instruments Corp., Austin, TX, USA) with 24-bit precision and a sampling rate of 10 kHz.

Table 2 outlines the main descriptive parameters of all the datasets considered in this study. All signals underwent initial visual inspection to assess reasonable signal quality. Signals were considered to have a reasonable signal quality if both the systolic and diastolic complexes were sufficiently recognizable. Only dorso-ventral SCG components, cranio-caudal GCG components, and first derivatives of high-frequency FCG components (dHF-FCG) were analyzed in this study.

**Table 2:** Descriptive parameters of all datasets considered in the study

Dataset	Signals	Cohort size		Gender		Age (years)	Subjects conditions	Sampling rate
		Healthy	Pathological	Male	Female			
**#1** [77]	SCG + GCG	29	0	29	0	29 ± 5	Supine, quiet breathing	800 Hz
**#2** [78]	SCG	20	0	12	8	24.7 ± 3.9	Supine, quiet breathing, listening to music	5 kHz
**#3** [79]	SCG + GCG	0	100	59	41	68 ± 14	Supine, quiet breathing	256 Hz
**#4** [41]	dHF-FCG	6	0	4	2	36.6 ± 11.0	Supine, quiet breathing	10 kHz

### Signals pre-processing

**Fig. 1:**
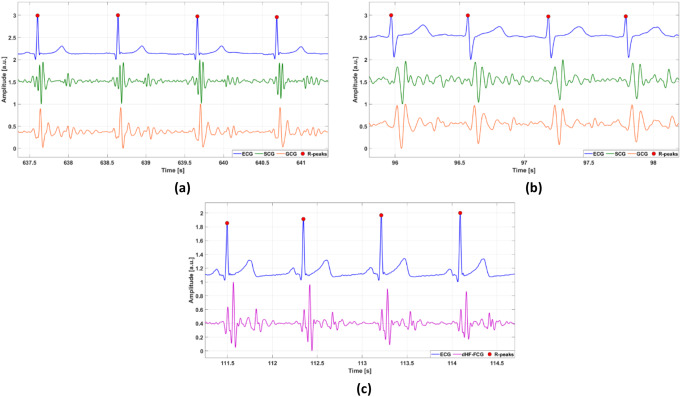
Excerpts of pre-processed signals: (**a**) ECG, SCG, and GCG signals of a healthy subject (subject #11 from dataset #1); (**b**) ECG, SCG, and GCG signals of a pathological subject (subject #CP16 from dataset #3); (**c**) ECG and dHF-FCG signals of a healthy subject (subject #4 from dataset #4). R peaks were marked with full red circles on ECG signals

Recordings from dataset #3 were oversampled at 1 kHz via linear interpolation to improve temporal resolution, by using the MATLAB^®^ function “*interp1*”. Afterwards, a 4th order zero-lag Butterworth band-pass filter with cut-off frequencies of 7 and 30 Hz was used to extract the high-frequency components of all SCG and GCG signals considered in this study. As in [44], the respiratory components were first extracted from raw FCG sensor signals via a Savitzky-Golay filter with order 3 and frame length approximately corresponding to a time interval of 1.5 s, and then subtracted from the raw signals to isolate the cardiac component. The resulting FCG signal was band-pass filtered in the 7–30 Hz frequency band via a 4th order zero-lag Butterworth filter to extract the high-frequency component (HF-FCG). Then, the first derivative (dHF-FCG) was computed as finite forward difference. On the other hand, all ECG signals were first band-pass filtered in the 0.5–40 Hz frequency band via a 4th order zero-lag Butterworth filter to remove baseline oscillations and high-frequency noises. Then, the 50 Hz powerline interference and its higher harmonics were removed via notch filters. Finally, the well-known Pan-Thompkins algorithm [8], implemented in the “BioSigKit” MATLAB^®^ toolbox [80], was used to obtain the temporal locations of R peaks. All the processing operations described in this study were carried out in MATLAB^®^ R2023b (MathWorks, Inc., Natick, MA, USA). Figure 1 shows some excerpts of pre-processed signals.

### Automatic template selection

**Fig. 2:**
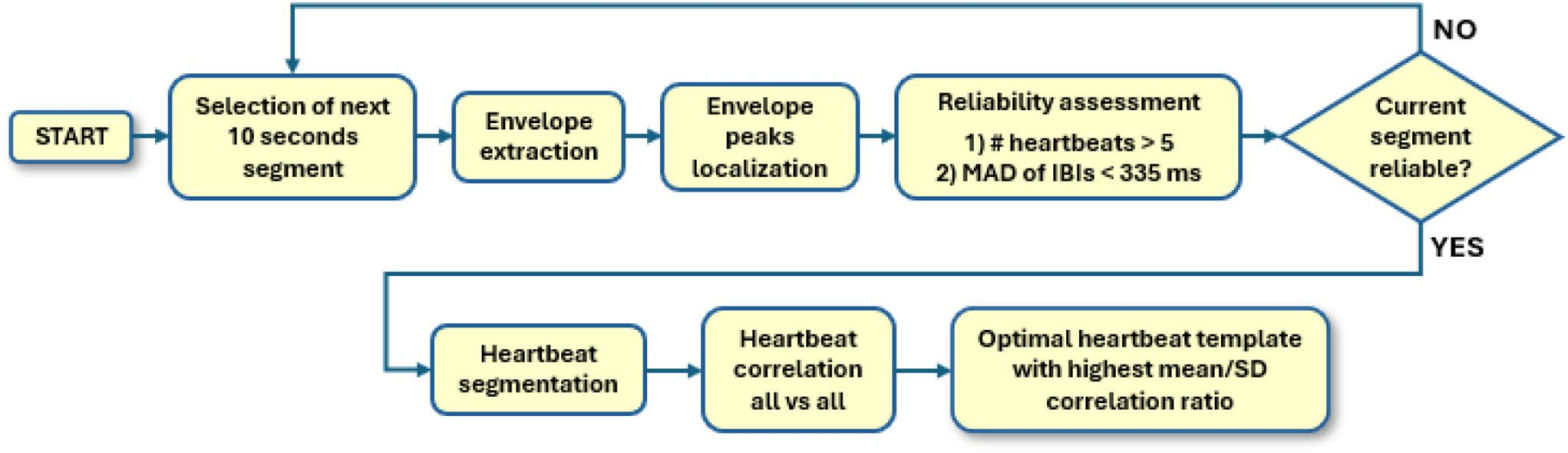
Flow diagram of the automatic template selection algorithm

After the pre-processing stage, heartbeat detection based on template matching was applied to all cardiomechanical signals. The first step of the template matching method is template selection. In this study, a novel automatic template selection algorithm was proposed. A flow diagram that describes the automatic template selection algorithm is depicted in Fig. 2.

#### Identification of a reliable window for template selection

The algorithm considers a first-time window of ten seconds at the beginning of the recording and generates an envelope signal by computing the 4th power of the signal, which is then low-pass filtered at 3 Hz via a 2nd order zero-lag Butterworth filter. The amplitude of the envelope signal thus obtained is normalized to the absolute maximum value of the signal in the 10-second segment. Afterwards, the peaks of the envelope signal, which correspond to individual heartbeats, are located via the MATLAB^®^ function “*findpeaks*”, by setting a minimum peak prominence of 0.25, and their time locations are used to obtain IBIs estimates. However, disturbances in signal measurement may mask the envelope peaks corresponding to heartbeats or produce spurious peaks that do not correspond to actual heartbeats. For this reason, before proceeding with template selection, the algorithm evaluates the reliability of the current 10-second segment according to two criteria: a) a minimum number of heartbeats should have been detected in the current segment; (b) the variability of the IBIs should be lower than a reasonable threshold. The first criterion is aimed at ruling out segments where too many heartbeats were missed; the second criterion is aimed at ruling out segments where too many artifacts produced spurious envelope peaks. The minimum number of heartbeats was set to 5, by assuming a reasonable minimum heart rate of 30 bpm. The variability of IBIs could be ascribed both to spurious peaks and to the intrinsic variability of the heart rhythm, e.g., due to arrythmias. Atrial fibrillation causes the highest variability in heart rhythm, and according to [81], the standard deviation of inter-beat intervals (SDNN) in patients with atrial fibrillation is 117.9 ± 72.2 ms, which means that the 99% of SDNN values are lower than 334.5 ms (assumption of normal distribution). A higher SDNN is more likely to be due to spurious envelope peaks. However, SDNN computed on a small number of IBIs, as in the 10-second window considered for template selection, can be easily corrupted by outliers. For this reason, the mean absolute deviation (MAD) was chosen as a more robust measure of IBIs variability, and the threshold for the MAD of IBIs was set to 335 ms. Hence, a 10-second segment is considered reliable if at least 5 heartbeats have been identified on the envelope signal, and the MAD of the IBIs is lower than 335 ms. If the algorithm considers the current segment not reliable, it jumps to the next 10-second segment and repeats the same evaluation.

#### Template identification within the reliable window

**Fig. 3:**
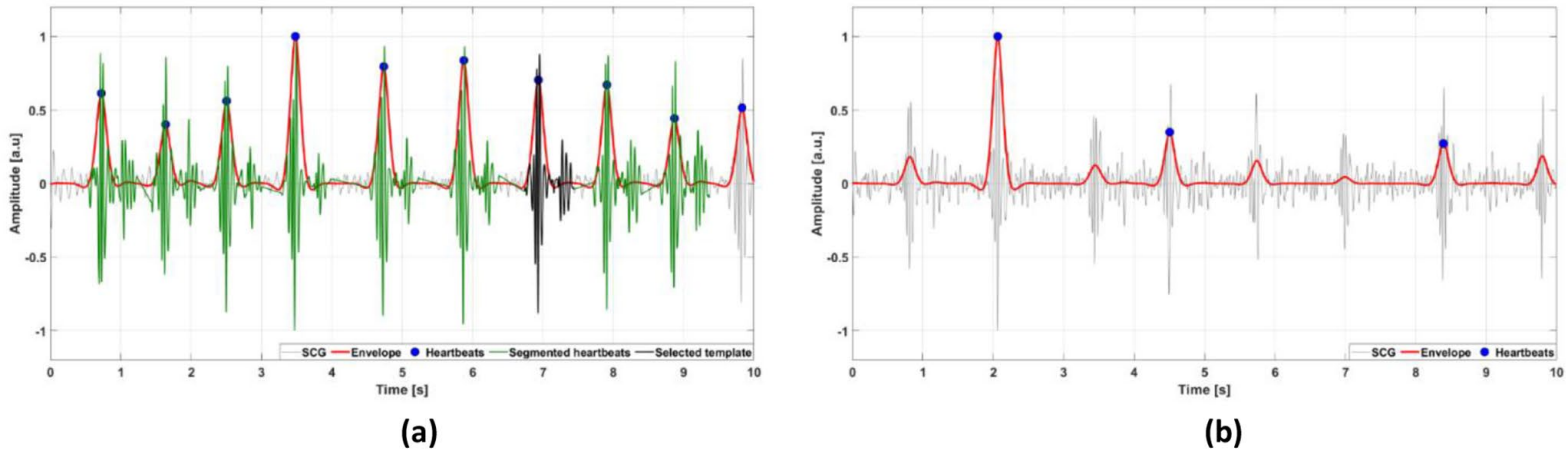
Examples of 10-second segments of SCG signals classified by the automatic template selection as: (**a**) reliable; (**b**) non-reliable. The SCG signals are depicted as thin grey lines, the envelope signals as thick red lines, the locations of heartbeats (envelope peaks) are marked with full blue circles, the segmented heartbeats are depicted as thick green lines, and the selected template as a thick black line. The segment in panel (**b**) was classified as non-reliable because only 3 heartbeats were identified: therefore, no segmented heartbeats and template are shown in this panel

**Fig. 4:**
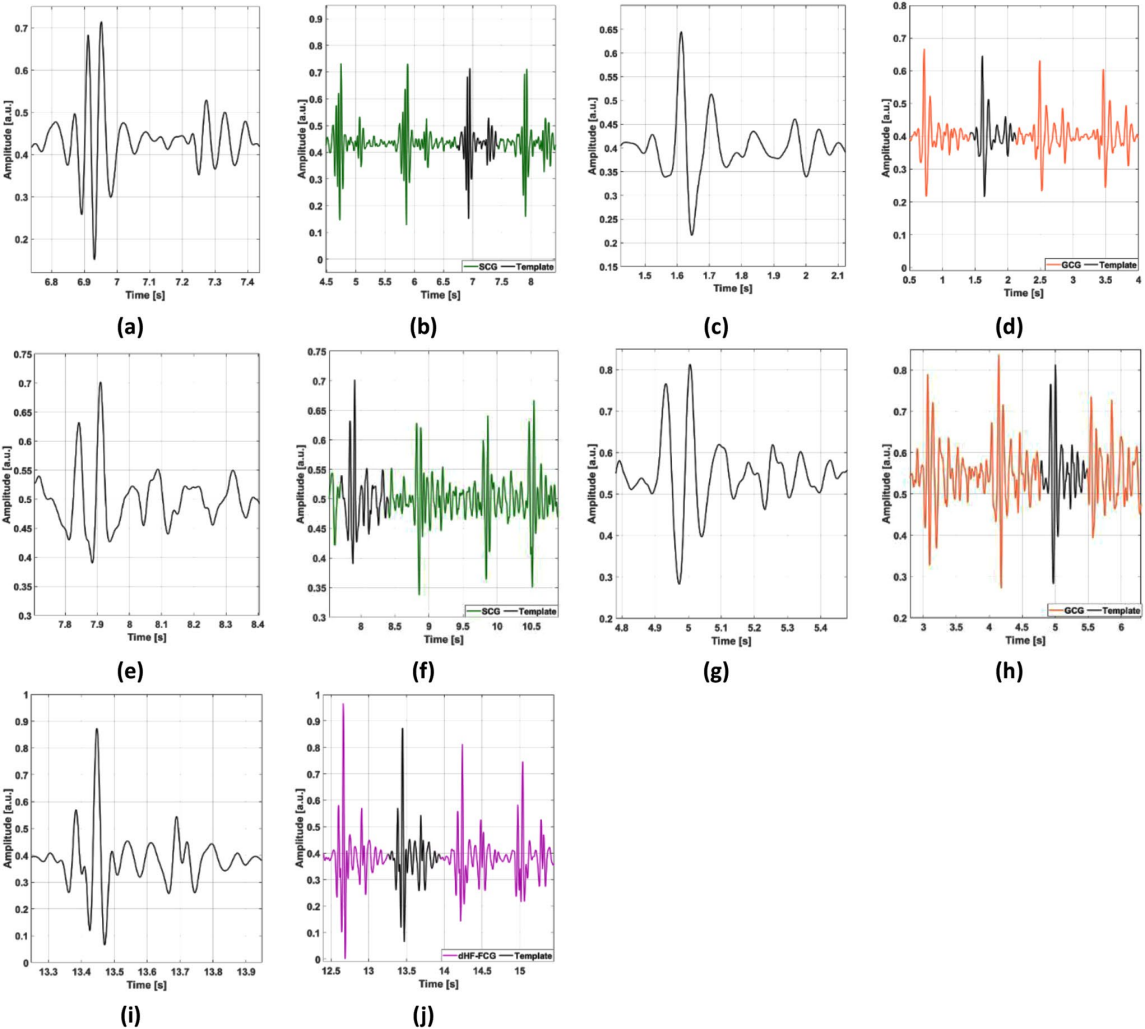
Examples of templates selected in SCG, GCG, and dHF-FCG signals. (**a**) and (**c**) depict the templates selected in the SCG and GCG signals of subject #11 (dataset #1, healthy subjects); (**b**) and (**d**) show some excerpts of SCG and GCG signals from which the templates in (**a**) and (**c**) were extracted; (**e**) and (**g**) depict the templates selected in the SCG and GCG signals of subject #CP16 (dataset #3, pathological subjects); (**f**) and (**h**) show some excerpts of SCG and GCG signals from which the templates in (**e**) and (**g**) were extracted; (**i**) depicts the template selected in the dHF-FCG of subject #4 (dataset #4, healthy subjects); (**j**) shows an excerpt of the dHF-FCG signal from which the template in (**i**) was extracted

When a reliable segment is found, the template selection proceeds by identifying potential candidates for the heartbeat template. The algorithm segments all detected heartbeats by considering time intervals from 200 ms before to 500 ms after each envelope peak. Each segmented heartbeat is a potential candidate for template matching, therefore a criterion for the selection of the best candidate was defined. The actual template is selected as the heartbeat segment with the highest average similarity and the lowest variability in similarity with all other heartbeats. This criterion ideally ensures that the selected template would find the highest number of heartbeat matches in the whole signal. To this purpose, the algorithm calculates the correlation coefficients of each heartbeat with all other heartbeats (all vs. all), and then computes the mean and the standard deviation (SD) of such correlation coefficients. The heartbeat segment with the highest ratio of mean correlation coefficient and SD of correlation coefficients is eventually selected as the heartbeat template. Figure 3 shows some examples of 10-second segments classified as reliable (Fig. 3.a) and non-reliable (Fig. 3.b) by the algorithm. Figure 4 shows examples of templates selected in SCG and GCG signals from healthy subjects, SCG and GCG signals from pathological subjects, and dHF-FCG signals from healthy subjects.

### Template matching method for heartbeat detection

**Fig. 5:**
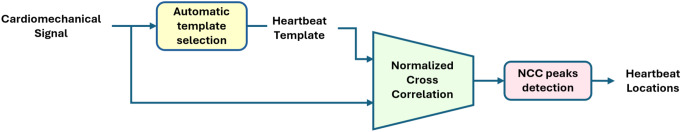
Flow diagram of the fully automated template matching method

The automatic template selection algorithm described in Sect. 2.3 is integrated into a template matching method that consists of three steps: (1) template selection; (2) computation of normalized cross-correlation (NCC) function between the selected template and the whole signal; (3) localization of peaks on NCC. A flow diagram that describes the whole template matching method is depicted in Fig. 5.

NCC is used as a quantitative measure of local similarity of the signal to the selected template. Therefore, NCC peaks correspond to signal chunks that mostly match the heartbeat template and provide the temporal locations of the heartbeats in the signal. NCC peaks are located via the MATLAB^®^ function “*findpeaks*”, by setting a minimum peak prominence of 0.5 and a minimum peak distance of 0.5 s. The fully automated template matching method was implemented as a MATLAB^®^ function [76]. The function only requires a cardiomechanical signal and its sampling rate as inputs, and provides the user with temporal locations of heartbeats within the signal. Additionally, the function allows the user to change its parameters for custom use. The fully automated template matching method was successfully applied to all 256 cardiomechanical signals considered in this study. Figure S1 in the supplementary materials shows some examples of its performance in heartbeat detection on SCG, GCG, and dHF-FCG signals. Inter-beat intervals were also estimated as temporal differences between consecutive heartbeats located in cardiomechanical signals, while reference IBIs were obtained from ECG recordings as temporal differences between consecutive R peaks, as shown in Figure S2 in the supplementary materials.

### Performance assessment via statistical analyses

The accuracy of the temporal locations of heartbeats provided by the proposed template matching method was assessed by comparison with simultaneously acquired ECG recordings, which were considered as the ground truth for heartbeat detection. This allowed the detection of missed or misidentified heartbeats. To this end, the NCC peaks localized by the proposed method were classified as either true positives (TPs) or false positives (FPs). Specifically, multiple NCC peaks within one cardiac cycle, or single NCC peaks occurring at temporal positions unlikely to correspond a heartbeat, were considered as FPs. In addition, cardiac cycles where no NCC peaks had been identified were considered as false negatives (FNs). Statistical analyses were carried out on identified heartbeats and IBIs to assess the performance of the proposed template matching method. In particular, to assess the performance of heartbeat detection, sensitivity and positive predictive value (PPV) were computed. Finally, to evaluate the accuracy of the temporal locations of heartbeats, the IBIs estimated from cardiomechanical signals were compared with those obtained from reference ECG signals via linear regression, correlation, and Bland-Altman analyses. The IBIs related to the FP and FN were excluded from the analyses. The Matlab^®^ function “*bland-altman-and-correlation-plot*” [82] was used for this purpose.

## Results

### Results on SCG and GCG signals of healthy subjects

**Fig. 6:**
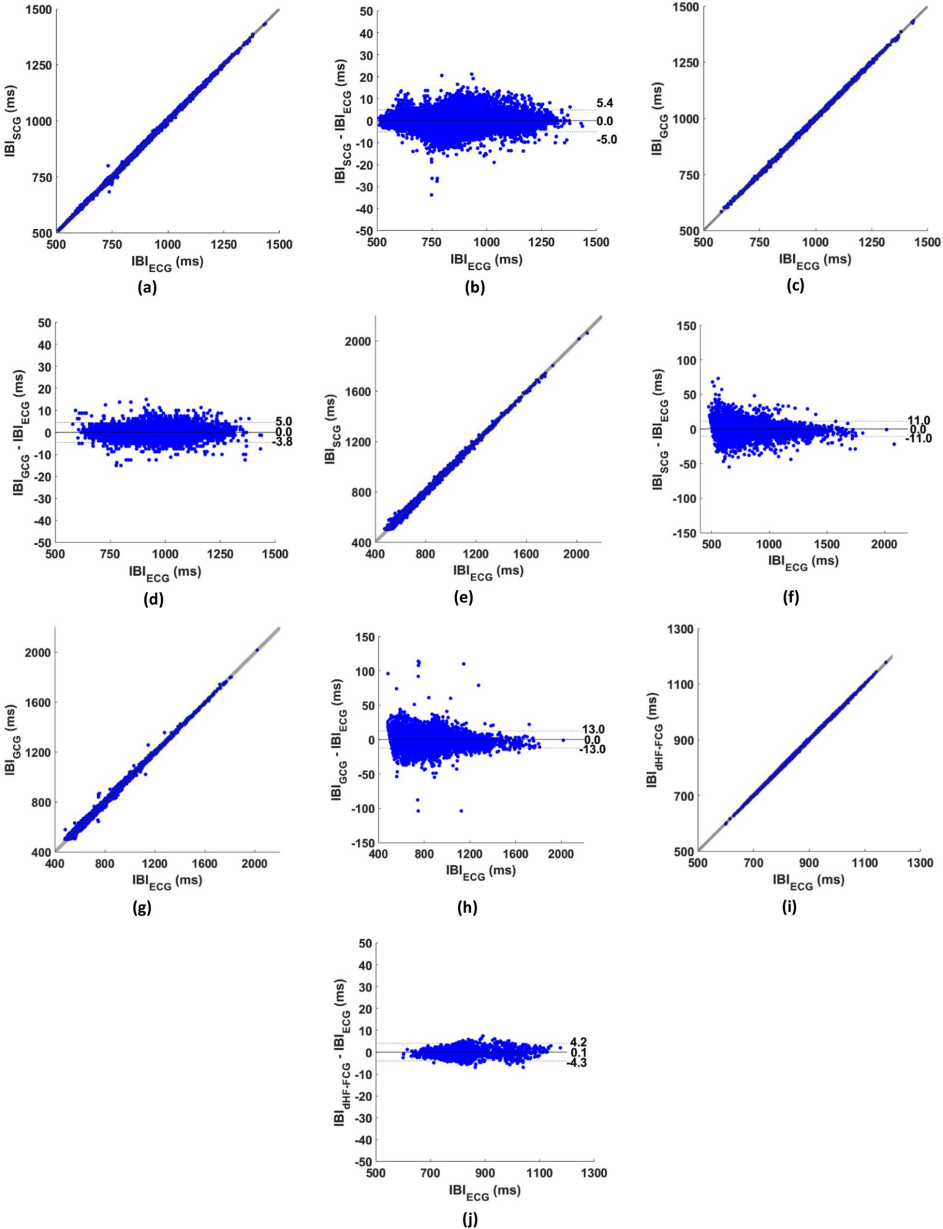
Results of statistical analyses performed on SCG, GCG, and dHF-FCG signals. linear regression and correlation plots are shown in the left column, while Bland-Altman plots in the right column. (**a**) and (**b**) show the results obtained on SCG signals of healthy subjects (datasets #1 and #2); (**c**) and (**d**) show the results obtained on GCG signals of healthy subjects (datasets #1 and #2); (**e**) and (**f**) show the results obtained on SCG signals of pathological subjects (dataset #3); (**g**) and (**h**) show the results obtained on GCG signals of pathological subjects (dataset #3); (**i**) and (**j**) show the results obtained on dHF-FCG signals of healthy subjects (dataset #4)

SCG data of healthy subjects from datasets #1 and #2 were analyzed together. On these data, the proposed method correctly identified 78,212 heartbeats out of a total of 79,937 reference heartbeats, thus achieving a sensitivity and PPV of 97.8% and 96.7%, respectively. On GCG signals of healthy subjects (dataset #1), 11,804 heartbeats were identified out of a total of 12,259 reference heartbeats, which yielded a sensitivity of 96.3% and a PPV of 94.5%. Results achieved across all subjects are outlined in Table 3, while results achieved per single subject are reported in Tables S1, S2, and S3 in the supplementary materials. Linear regression and correlation analyses were performed on 77,239 and 11,605 IBIs, respectively for SCG and GCG, and reported practically unit slopes and negligible intercepts, with R^2^ values in excess of 0.999, for both SCG and GCG. Bland-Altman analyses reported null biases with limits of agreement (LoA) of [-5.0; 5.4] ms for SCG and [-3.8; 5.0] ms for GCG. The results of linear regression, correlation, and Bland-Altman analyses are depicted in panels (a-d) of Fig. 6 and outlined in Tables 4 and 5.

### Results on SCG and GCG signals of pathological subjects

On SCG data of pathological subjects (dataset #3) the proposed method correctly identified 28,342 heartbeats out of a total of 33,265 reference heartbeats, thus achieving a sensitivity and PPV of 85.2% and 95.1%, respectively. On GCG signals of pathological subjects (dataset #3), 34,516 heartbeats were identified out of a total of 40,527 reference heartbeats, which yielded a sensitivity of 85.2% and a PPV of 94.9%. Results achieved across all subjects are outlined in Table 3, while results achieved per single subject are reported in Tables S4 and S5 in the supplementary materials. Linear regression and correlation analyses were performed on 25,359 and 30,765 IBIs, respectively for SCG and GCG, and reported almost unit slopes and negligible intercepts, with R^2^ values in excess of 0.998, for both SCG and GCG. Bland-Altman analyses reported null biases with LoA of ± 11 ms for SCG and ± 13 ms ms for GCG. The results of linear regression, correlation, and Bland-Altman analyses are depicted in panels (e-h) of Fig. 6 and outlined in Tables 4 and 5.

### Results on FCG signals of healthy subjects

The proposed method correctly identified 1422 heartbeats out of a total of 1434 reference heartbeats in dHF-FCG signals of healthy subjects (dataset #4), thus achieving a sensitivity of 99.2% and a PPV of 99.3%. Results achieved across all subjects are outlined in Table 3, while results achieved per single subject are reported in Table S6 in the supplementary materials. Linear regression and correlation analyses were performed on 1405 IBIs, and reported practically unit slopes and negligible intercepts, with R^2^ values in excess of 0.999. Bland-Altman analyses reported a negligible bias of 0.1 ms with LoA of [-4.3; 4.2] ms. The results of linear regression, correlation, and Bland-Altman analyses are depicted in panels (i, j) of Fig. 6 and outlined in Tables 4 and 5.

**Table 3:** Performances of heartbeat detection on 256 cardiomechanical signals from all datasets

Health conditions	Signal	*N*_Subjects_	*N*_Heartbeats_	Sensitivity (%)	PPV (%)
Healthy	SCG	49	79,937	97.8	96.7
	GCG	29	12,259	96.2	94.6
	dHF-FCG	6	1434	99.2	99.3
Pathological	SCG	77	33,265	85.2	95.1
	GCG	95	40,527	85.2	94.9

**Table 4:** Results of linear regression and correlation analyses performed on IBIs extracted from 256 cardiomechanical signals from all datasets

Health conditions	Signal	*N*_Subjects_	*N*_IBI_	Slope	CI_Slope_	Intercept (ms)	Ci_intercept_ (ms)	*R* ^2^	CI_R2_
Healthy	SCG	49	77,239	1.002	[1.002; 1.002]	-1.5	[-1.6; -1.3]	0.9996	[0.9996; 0.9996]
	GCG	29	11,605	1.002	[1.001; 1.002]	-1.4	[-1.7; -1.1]	0.9997	[0.9997; 0.9997]
	dHF-FCG	6	1405	1.001	[1.000; 1.002]	-0.8	[-1.6; 0.1]	0.9996	[0.9996; 0.9996]
Pathological	SCG	77	25,359	0.997	[0.996; 0.997]	3.0	[2.7; 3.3]	0.9989	[0.9989; 0.9989]
	GCG	95	30,765	0.997	[0.996; 0.997]	3.0	[2.7; 3.4]	0.9986	[0.9986; 0.9986]

**Table 5:** Results of Bland-Altman analyses performed on IBIs extracted from 256 cardiomechanical signals from all datasets. For measurement differences with non-normal distribution, the bias was computed as the median of the differences, and LoAs were computed as 2.5th and 97.5th percentiles of the differences

Health conditions	Signal	N_Subjects_	N_IBI_	BIAS (ms)	CI_BIAS_ (ms)	LoA (ms)	CI_LOA_MIN_ (ms)	CI_LOA_MAX_ (ms)
Healthy	SCG	49	77,239	0.0	[0.0; 0.0]	[-5.0; 5.4]	[-5.0; -5.0]	[5.4; 5.6]
	GCG	29	11,605	0.0	[0.0; 0.0]	[-3.8; 5.0]	[-5.0; -3.8]	[5.0; 5.0]
	dHF-FCG	6	1405	0.1	[0.0; 0.3]	[-4.3; 4.2]	[-4.6; -4.1]	[3.8; 4.3]
Pathological	SCG	77	25,359	0.0	[0.0; 0.0]	[-11.0; 11.0]	[-12.0; -11.0]	[11.0; 12.0]
	GCG	95	30,765	0.0	[0.0; 0.0]	[-13.0; 13.0]	[-13.0; -12.0]	[13.0; 14.0]

## Discussion

### Performance of the proposed method

The proposed fully automated template matching method for ECG-free heartbeat detection was validated on the largest subjects cohort of all studies on this topic, and also on the largest cohort of pathological subjects. Some SCG and GCG signals recorded from VHD patients (dataset #3) were excluded from the analysis due to extremely poor signal quality, which almost prevented visual identification of heartbeats. The novel automatic template selection algorithm ensured an effective selection of heartbeat templates in all 256 signals considered in this study. The proposed template matching method demonstrated high performance in heartbeat detection. On all healthy subjects, it scored sensitivity and PPV of 97.8% and 98.6% for SCG, of 96.2% and 94.4% for GCG, and of 99.2% and 99.3% for FCG; on pathological subjects, it scored a sensitivity of 85% and a PPV of 95% for both SCG and GCG. The lower performances achieved on pathological subjects data were expected, because cardiomechanical signals of patients affected by cardiac diseases are known to exhibit non-conventional and highly instable morphologies [48]. The proposed method also demonstrated very high accuracy in the estimation of inter-beat intervals, as it achieved very good agreement with IBIs provided by the reference ECG, with non-significant biases, LoA within ± 6 ms for healthy subjects, and LoA within ± 13 ms for pathological subjects. Normalized cross-correlation is recognized to be a powerful similarity measure with high robustness to noise, able to recognize specific waveforms in noisy recordings. The results of this study highlight that the normalized cross-correlation can be advantageously used for recognizing the weak rhythmic vibrations (i.e. SCG and GCG) generated by heartbeats that generally have poor signal-to-noise ratio. This suggests that this technique may be useful for the recognition of other rhythmic biomedical signals such as sphygmic waves, blood flows, respiratory acts, etc. This could potentially lead to improvements in continuous and pervasive monitoring such as that performed by smartwatches or smart rings via the photoplethysmographic signal.

### Performance comparison with existing methods

The proposed method was compared with all existing studies on ECG-free heartbeat detection in SCG and GCG signals. Tables 6, 7 and 8 provide a quantitative comparison between the methods. Most of the studies have not declared the number of heartbeats analyzed; the others reported much smaller numbers of heartbeats as compared to the present study. Only five studies on SCG [46, 56, 60, 63, 66] reported similar or higher sensitivity and PPV with respect to the present study (Table 6): two of these [60, 66] do not declare the number of heartbeats analyzed, other two [46, 56] considered a significantly lower number of heartbeats, and only [63] considered a reasonably high number of heartbeats, although lower than the present study. Four studies on GCG [46, 56, 57, 60] presented similar or higher sensitivity and PPV with respect to the present study (Table 7): one of these [60] does not declare the number of heartbeats analyzed, while the other three [46, 56, 57] considered a significantly lower number of heartbeats. In terms of accuracy of IBIs or instantaneous HRs, the present study outperforms all existing studies on both SCG and GCG signals. Table 8 compares the performances achieved by the considered methods on SCG and GCG signals of pathological subjects, by using the same metrics of Tables 6 and 7. Only two studies considered pathological subjects [59, 60]: these have been carried out on significantly smaller cohorts of patients, and none of them declared the number of heartbeats analyzed. Even for pathological subjects, the present study outperforms all existing studies in terms of accuracy of IBIs or instantaneous HRs. The lower performances achieved by other methods could be ascribed to their heartbeat detection approaches based on the identification of specific markers in SCG/GCG signals. For example, the highest peak of the SCG signal for each heartbeat is considered as a temporal marker: it is typically located immediately after the R wave of the ECG and corresponds to the aortic valve opening. However, physiological variations of SCG morphology mean that this absolute maximum can shift between adjacent peaks of the same vibration, generating uncertainties about the location of a single beat. Other secondary temporal landmarks on the SCG signal are not always clearly recognizable too. The high inter-beat variability typical of SCG signals from pathological subjects makes the search for accurate temporal landmarks even more challenging and prone to error. In contrast, normalized cross-correlation evaluates SCG ensemble similarity over the entire heartbeat duration, therefore, it is much more robust with respect to the inevitable signal variations and the noise. It is also worth noting that even approaches based on artificial intelligence have achieved lower performances as compared to the proposed method. This suggests that capturing the complexity of cardiomechanical signals is difficult even for complex and powerful artificial intelligence algorithms.

### Limitations of the study

This study has some limitations. The performance of the proposed fully automated template matching method for ECG-free heartbeat detection was tested only on patients affected by VHDs, so the performance of the method on patients with different cardiac diseases was not evaluated. Moreover, data was collected only from patients in the same posture. It has previously been reported in literature that body posture influences the morphology of cardiomechanical signals [49], therefore the impact of posture changes was not considered in this study. Finally, the signals analyzed in this study had been acquired from patients at rest, thus preventing a thorough assessment of the robustness of the proposed method to motion artifacts caused by physical activities.

### Future developments

A more comprehensive assessment on patients affected by different cardiac diseases should be performed to confirm the potential of the proposed method to improve patients follow-up in a broad range of cardiac diseases via continuous, long-term, cardiomechanical monitoring. The proposed heartbeat detection method could also be further tested on signals recorded from patients in different postures. Particularly, an investigation is required to assess if a heartbeat template selected from a signal acquired from a subject in a certain posture is effective in detecting heartbeats on signals acquired from the subject in different postures. Future studies could evaluate the performance of the proposed heartbeat detection method on signals acquired from subjects during physical activities, to verify its suitability for continuous monitoring of subjects during activities of daily living, as well as athletes during sport performances.

**Table 6:** Comparison of studies on ECG-free heartbeat detection in SCG and GCG signals: performances on SCG signals acquired from healthy subjects

Study	Cohort size	Heartbeat detection performance			Accuracy of IBI/HR				
		Number of beats	Sensitivity	PPV	Slope	Intercept	*R* ^2^	BIAS	LOA
**Current study**	49	79,937	97.8	98.6	1	-1.5	0.999	0	[-5.0; 5.4] ms
D’Mello et al. [46]	25	23,984	96.6	99.7	N/A	N/A	0.9482	0.03 bpm	± 8.36 bpm
Hurnanen et al., 2018 [56]	66	8870	98.6	99.3	N/A	N/A	N/A	N/A	N/A
Tadi et al., 2016 [57]	30	9213	95.8	96.0	1	-0.872 ms	0.9993	0.08 ms	[-6.5; 6.6] ms
Milena et al. 2023 [58]	21	N/A	N/A	N/A	N/A	N/A	0.98	-0.15 ms	± 33.71 ms
Kaisti et al., 2019 [60]	29	N/A	99.9	99.6	N/A	N/A	N/A	0 ms	± 11 ms
Jia et al., 2015 [61]	10	N/A	N/A	N/A	N/A	N/A	N/A	N/A	N/A
Choudary et al., 2021 [62]	20	N/A	N/A	N/A	1	-3.03 ms	0.98	-0.24 ms	± 34 ms
Zheng et al., 2024 [63]	20	69 221	99.6	98.9	N/A	N/A	N/A	N/A	N/A
Scarpetta et al., 2022 [64]	8	N/A	N/A	N/A	N/A	N/A	N/A	N/A	N/A
Wahlstrom et al., 2017 [65]	66	N/A	N/A	N/A	N/A	N/A	N/A	N/A	± 16 ms
Cocconcelli et al., 2020 [66]	20	N/A	98.5	98.6	N/A	N/A	99.3	0 ms	± 9.2 ms
	13	N/A	99.1	97.9	N/A	N/A	98.4	0 ms	± 12.4 ms
Schipper et al., 2024 [67]	147	N/A	79.3	98	N/A	N/A	N/A	-0.35 bpm	[-1.7; +1] bpm
Pickert et al., 2024 [68]	12	N/A	97.6	N/A	N/A	N/A	N/A	N/A	N/A
Thakkar & Sahoo, 2020 [69]	20	9000	93	94	N/A	N/A	N/A	N/A	N/A
Chen et al., 2021 [70]	20	N/A	97	98	N/A	N/A	N/A	0 bpm	± 1.3 bpm
Duraj et al., 2022 [71]	20	35,973	96.7	96.9	N/A	N/A	N/A	N/A	N/A

**Table 7:** Comparison of studies on ECG-free heartbeat detection in SCG and GCG signals: performances on GCG signals acquired from healthy subjects

Study	Cohort Size	Heartbeat detection performance			Accuracy of IBI/HR				
		Number of beats	Sensitivity	PPV	Slope	Intercept	*R* ^2^	BIAS	LOA
**Current study**	29	12,259	96.2	94.4	1	-1.4	0.999	0	[-3.8; 5.0] ms
D’Mello et al. [46]	25	23,984	96.6	99.7	N/A	N/A	0.931	0.5 bpm	± 9.79 bpm
Hurnanen et al., 2018 [56]	66	8870	99.1	99.7	N/A	N/A	N/A	N/A	N/A
Tadi et al., 2016 [57]	30	9213	95.8	96	1	-0.872 ms	0.999	0.08 ms	[-6.5; 6.6] ms
Milena et al. 2023 [58]	21	N/A	N/A	N/A	N/A	N/A	0.98	-0.13 ms	± 26.43 ms
Kaisti et al., 2019 [60]	29	N/A	99.9	99.6	N/A	N/A	N/A	0	± 11 ms
Jia et al., 2015 [61]	10	N/A	N/A	N/A	N/A	N/A	N/A	N/A	N/A

**Table 8:** Comparison of studies on ECG-free heartbeat detection in SCG and GCG signals: performances on SCG and GCG signals acquired from pathological subjects

Study	Signal	Cohort Size	Heartbeat detection performance			Accuracy of IBI/HR				
			Number of beats	Sensitivity	PPV	Slope	Intercept	*R* ^*2*^	BIAS	LOA
**Current study**	SCG	77	33,265	85.2	95.1	0.997	3	0.999	0	± 11 ms
	GCG	95	40,527	85.2	94.9	0.997	3	0.999	0	± 13 ms
Tadi et al., 2019 [59]	SCG	19	N/A	84	81	N/A	N/A	N/A	N/A	N/A
	GCG	19	N/A	87	84	N/A	N/A	N/A	N/A	N/A
	SCG + GCG	19	N/A	94	93	N/A	N/A	N/A	N/A	N/A
Kaisti et al., 2019 [60]	SCG	12	N/A	92.8	91.7	N/A	N/A	N/A	N/A	N/A
	GCG	12	N/A	93.3	92.3	N/A	N/A	N/A	N/A	N/A
	SCG + GCG	12	N/A	96.1	95.6	N/A	N/A	N/A	0.5 ms	± 113.5 ms

## Conclusions

This study presented a fully automated template matching method for ECG-free heartbeat detection in cardiomechanical signals. The proposed method is an advancement of an earlier method, whose main limitation was the need to manually select the heartbeat template to search within the signal [72, 73, 74, 75]. The automatic template selection algorithm proposed in this study overcomes this limitation, thus making the overall method fully automated, reproducible, and operator-independent. This study represents an important step toward the effective implementation of continuous, long-term, ECG-free patient monitoring via wearable cardiomechanical sensors. The performance validation carried out in this study showed that, beyond the very high performance achieved on healthy subjects, the proposed method also ensured high performance on pathological subjects, thus demonstrating its effectiveness in monitoring actual patients, even with irregular and non-repetitive heartbeat morphologies. This is an often underrated aspect, as almost none of the existing ECG-free heartbeat detection methods described in the literature have ever been validated on data from real patients with cardiac diseases. The proposed method outperforms all existing methods in terms of accuracy of inter-beat intervals, both on healthy and pathological subjects. Hence, it represents the current state of the art. It is also important to highlight that many of the existing methods are based on complex and computationally-intensive algorithms, for example, based on artificial intelligence, while the template matching method proposed in this study ensures high performance by requiring low computational resources, like those offered by low-performing microcontrollers. These features make the proposed method an ideal candidate for the implementation on wearable and personal devices equipped with inertial sensors or force sensors, with low power consumption for extended operating time. This could enable continuous, long-term, ECG-free monitoring of subjects for early detection of pathological signs, such as atrial fibrillation and other arrythmias, valvular pathologies, ominous signs of congestive heart failure.

## Electronic Supplementary Material

Below is the link to the electronic supplementary material.

Supplementary materials

## Data Availability

Data considered in this study is available in public repositories, apart from a small dataset that is available upon request to Emilio Andreozzi. The software that implements the method proposed in this study is available in a public repository as well.
