# Supplementary material for: Fully automated template matching method for ECG-free heartbeat detection in cardiomechanical signals of healthy and pathological subjects
Source: Phys Eng Sci Med. 2025 Mar 13;48(2):649–64. doi: 10.1007/s13246-025-01531-3 (PMC12208961; doi:10.1007/s13246-025-01531-3)
Supplement: Supplementary file 1 — Supplementary materials [file 13246_2025_1531_MOESM1_ESM.docx]

# **PHYSICAL AND ENGINEERING SCIENCES IN MEDICINE**

# Fully Automated Template Matching Method for ECG-Free Heartbeat Detection in Cardiomechanical Signals of Healthy and Pathological Subjects

Salvatore Parlato^1^ (0009-0000-9314-5126), Jessica Centracchio^1,*^ (0000-0003-3422-8727), Daniele Esposito^2^ (0000-0003-0716-8431), Paolo Bifulco^1^ (0000-0002-9585-971X), Emilio Andreozzi^1^ (0000-0003-4829-3941)

^1^ Department of Electrical Engineering and Information Technologies, University of Naples Federico II, Via Claudio, 21, Naples, I-80125, Italy

^2^ Department of Information and Electrical Engineering and Applied Mathematics, University of Salerno, Via Giovanni Paolo II, 132, Fisciano, I-84084, Italy

^*^ corresponding author: jessica.centracchio@unina.it

| 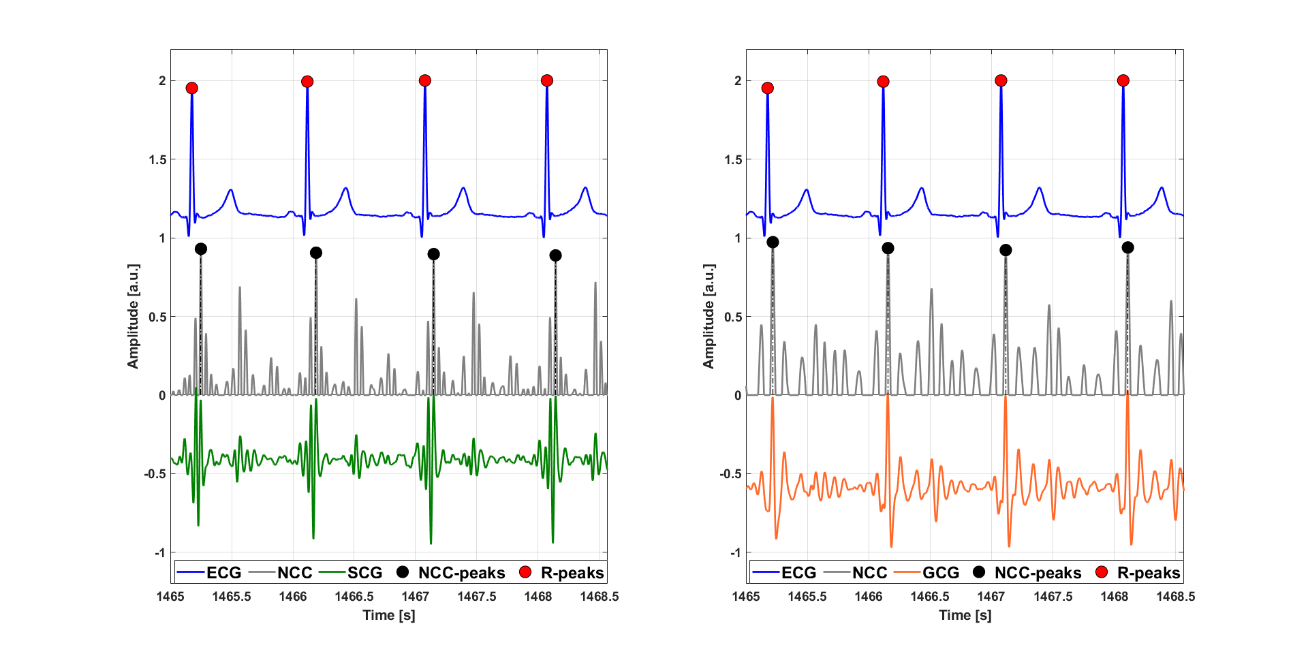 |
| --- |
| **(a)** |
| 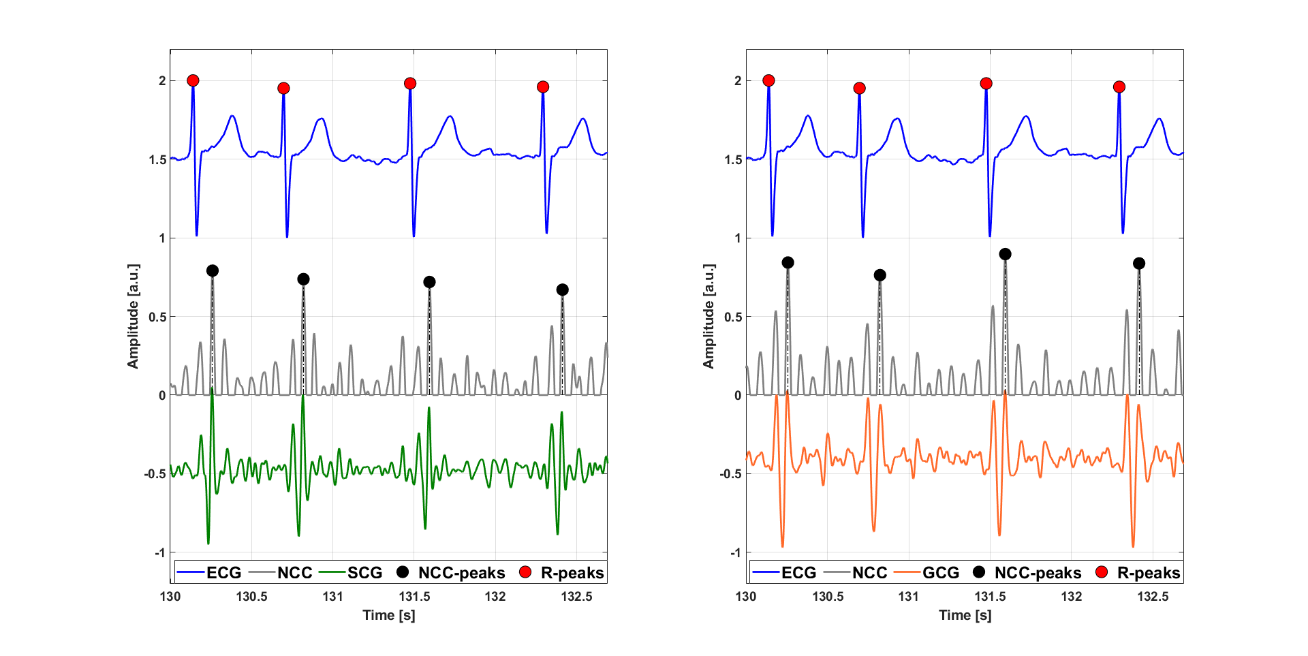 |
| **(b)** |
| 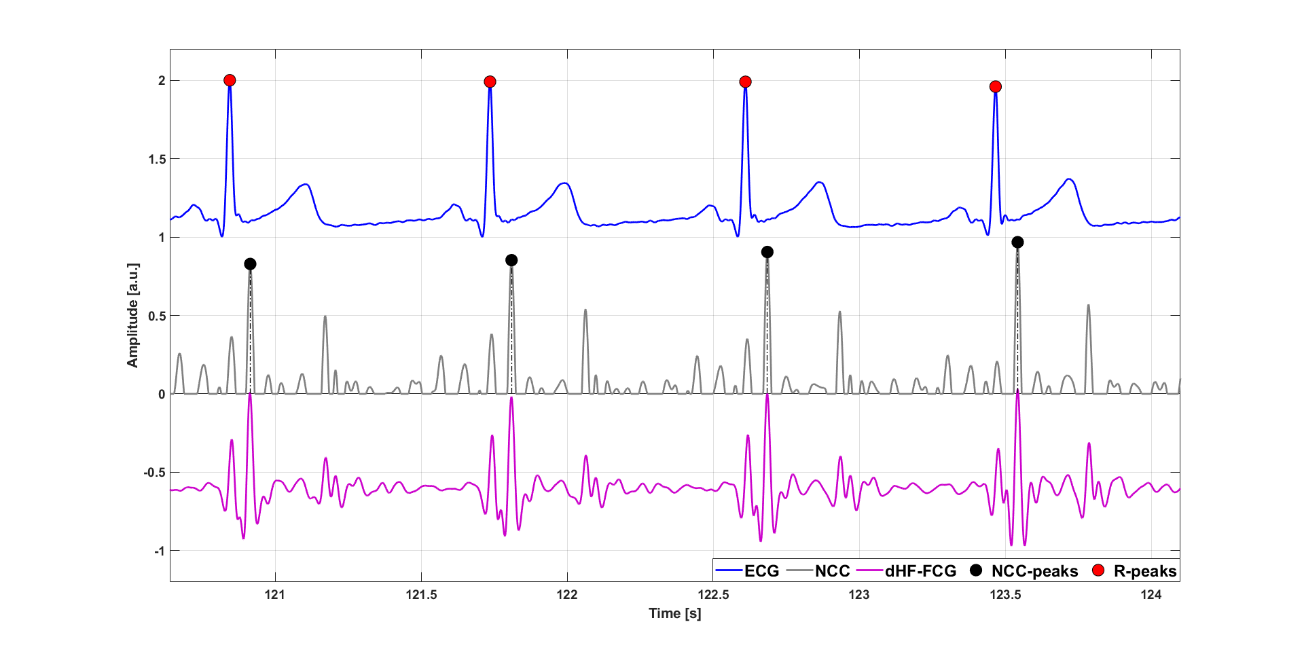 |
| **(c)** |

**Fig. S1.** Examples of heartbeat localization performed with the proposed method. (a) Heartbeats detected in SCG (left) and GCG (right) signals of a healthy subject (subject #11 from dataset #1); (b) heartbeats detected in SCG (left) and GCG (right) signals of a pathological subject (subject #CP-16 from dataset #3); (c) heartbeats detected in dHF-FCG signals of a healthy subject (subject #4 from dataset #4).


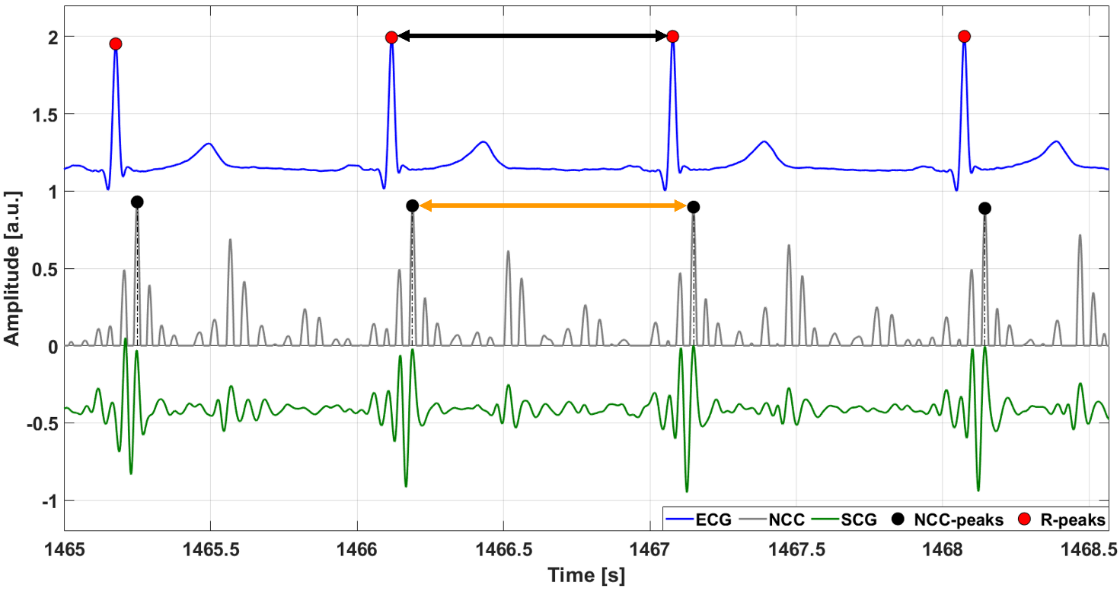


**Fig. S2.** Examples of IBIs estimation in ECG and SCG signals (subject #11 from dataset #1). A black double arrow depicts an IBI obtained from ECG as the time interval between two consecutive R-peaks. An orange double arrow depicts the corresponding IBI obtained from SCG as the time interval between two consecutive NCC peaks.

**Table S1.** Number of heartbeats detected in the ECG and SCG signals of 29 healthy patients from dataset #1, along with the number of FP, FN identified in SCG signals and the number of compared IBIs.

| **Patient**  **ID #** | **R-peaks** | **TP** | **FP** | **FN** | **IBI** |
| --- | --- | --- | --- | --- | --- |
| sub01 | 134 | 132 | 2 | 2 | 128 |
| sub02 | 153 | 121 | 11 | 32 | 118 |
| sub03 | 126 | 115 | 14 | 11 | 104 |
| sub04 | 167 | 157 | 4 | 10 | 152 |
| sub05 | 179 | 175 | 0 | 4 | 171 |
| sub06 | 142 | 108 | 33 | 34 | 104 |
| sub07 | 140 | 119 | 15 | 21 | 114 |
| sub08 | 294 | 293 | 0 | 1 | 290 |
| sub09 | 482 | 435 | 32 | 47 | 403 |
| sub10 | 569 | 568 | 0 | 1 | 565 |
| sub11 | 1490 | 1469 | 20 | 21 | 1446 |
| sub12 | 537 | 520 | 14 | 17 | 503 |
| sub13 | 493 | 493 | 0 | 0 | 492 |
| sub14 | 435 | 397 | 25 | 38 | 377 |
| sub15 | 573 | 431 | 93 | 142 | 376 |
| sub16 | 416 | 416 | 0 | 0 | 415 |
| sub17 | 208 | 199 | 3 | 9 | 192 |
| sub18 | 595 | 595 | 0 | 0 | 594 |
| sub19 | 408 | 403 | 6 | 5 | 397 |
| sub20 | 420 | 419 | 1 | 1 | 416 |
| sub21 | 489 | 487 | 2 | 2 | 483 |
| sub22 | 671 | 345 | 25 | 326 | 276 |
| sub23 | 184 | 184 | 0 | 0 | 183 |
| sub24 | 510 | 510 | 0 | 0 | 509 |
| sub25 | 492 | 490 | 1 | 2 | 486 |
| sub26 | 517 | 517 | 0 | 0 | 516 |
| sub27 | 642 | 641 | 0 | 1 | 640 |
| sub28 | 468 | 468 | 0 | 0 | 467 |
| sub29 | 332 | 246 | 86 | 86 | 197 |
| **Total** | **12266** | **11453** | **387** | **813** | **11114** |

**Table S2.** Number of heartbeats detected in the ECG and GCG signals of 29 healthy patients from dataset #1, along with the number of FP, FN identified in GCG signals and the number of compared IBIs.

| **Patient**  **ID #** | **R-peaks** | **TP** | **FP** | **FN** | **IBI** |
| --- | --- | --- | --- | --- | --- |
| sub01 | 134 | 134 | 2 | 0 | 132 |
| sub02 | 153 | 119 | 29 | 34 | 115 |
| sub03 | 126 | 110 | 21 | 16 | 105 |
| sub04 | 167 | 165 | 1 | 2 | 162 |
| sub05 | 179 | 179 | 0 | 0 | 178 |
| sub06 | 142 | 128 | 40 | 15 | 118 |
| sub07 | 140 | 136 | 4 | 4 | 131 |
| sub08 | 294 | 292 | 1 | 2 | 288 |
| sub09 | 482 | 432 | 49 | 50 | 398 |
| sub10 | 569 | 568 | 0 | 1 | 565 |
| sub11 | 1490 | 1478 | 19 | 12 | 1465 |
| sub12 | 537 | 532 | 6 | 5 | 526 |
| sub13 | 493 | 493 | 0 | 0 | 492 |
| sub14 | 435 | 254 | 157 | 181 | 229 |
| sub15 | 573 | 573 | 0 | 0 | 572 |
| sub16 | 416 | 415 | 1 | 1 | 412 |
| sub17 | 208 | 198 | 21 | 10 | 191 |
| sub18 | 595 | 595 | 1 | 0 | 593 |
| sub19 | 408 | 403 | 199 | 5 | 398 |
| sub20 | 420 | 420 | 1 | 0 | 418 |
| sub21 | 489 | 488 | 1 | 0 | 487 |
| sub22 | 671 | 643 | 14 | 28 | 636 |
| sub23 | 184 | 184 | 0 | 0 | 183 |
| sub24 | 510 | 510 | 1 | 0 | 508 |
| sub25 | 492 | 490 | 2 | 2 | 486 |
| sub26 | 517 | 517 | 10 | 0 | 515 |
| sub27 | 642 | 641 | 0 | 1 | 640 |
| sub28 | 468 | 467 | 15 | 1 | 464 |
| sub29 | 332 | 247 | 85 | 85 | 198 |
| **Total** | **12266** | **11811** | **680** | **455** | **11605** |

**Table S3.** Number of heartbeats detected on 20 long-lasting SCG and ECG signals presented in dataset #2, along with the number of FP, FN identified in GCG signals and the number of compared IBIs.

| **Patient**  **ID #** | **R-peaks** | **TP** | **FP** | **FN** | **IBI** |
| --- | --- | --- | --- | --- | --- |
| m001 | 2849 | 2738 | 111 | 111 | 2652 |
| m002 | 3235 | 3232 | 3 | 3 | 3227 |
| m003 | 3094 | 2993 | 99 | 101 | 2914 |
| m004 | 3427 | 3371 | 48 | 56 | 3322 |
| m005 | 3593 | 3554 | 26 | 39 | 3518 |
| m006 | 3116 | 3063 | 32 | 53 | 3034 |
| m007 | 2614 | 2599 | 14 | 15 | 2583 |
| m008 | 5018 | 4842 | 176 | 176 | 4779 |
| m009 | 3159 | 3079 | 85 | 80 | 3036 |
| m010 | 2988 | 2931 | 12 | 57 | 2880 |
| m011 | 3596 | 3579 | 5 | 17 | 3568 |
| m012 | 3991 | 3959 | 12 | 32 | 3925 |
| m013 | 3711 | 3708 | 0 | 3 | 3703 |
| m014 | 3411 | 3366 | 25 | 45 | 3327 |
| m015 | 3205 | 3204 | 1 | 1 | 3201 |
| m016 | 3861 | 3856 | 5 | 5 | 3849 |
| m017 | 3461 | 3457 | 2 | 4 | 3452 |
| m018 | 2755 | 2650 | 62 | 105 | 2588 |
| m019 | 3191 | 3188 | 1 | 3 | 3184 |
| m020 | 3396 | 3390 | 3 | 6 | 3383 |
| **Total** | **67671** | **66759** | **722** | **912** | **66125** |

**Table S4.** Number of heartbeats detected in the ECG and SCG signals of 77 VHD patients from dataset #3, along with the number of FP, FN identified in SCG signals and the number of compared IBIs.

| **Patient**  **ID #** | **R-peaks** | **TP** | **FP** | **FN** | **IBI** |
| --- | --- | --- | --- | --- | --- |
| CP-01 | 448 | 448 | 0 | 0 | 447 |
| CP-02 | 651 | 641 | 2 | 10 | 632 |
| CP-04 | 661 | 599 | 26 | 62 | 550 |
| CP-05 | 509 | 494 | 10 | 15 | 481 |
| CP-07 | 451 | 447 | 3 | 4 | 442 |
| CP-08 | 544 | 492 | 43 | 52 | 452 |
| CP-09 | 364 | 175 | 17 | 189 | 89 |
| CP-10 | 506 | 131 | 64 | 375 | 34 |
| CP-11 | 656 | 555 | 3 | 101 | 478 |
| CP-12 | 423 | 414 | 8 | 9 | 405 |
| CP-13 | 837 | 829 | 0 | 8 | 819 |
| CP-14 | 472 | 238 | 52 | 234 | 110 |
| CP-15 | 630 | 612 | 9 | 18 | 601 |
| CP-16 | 355 | 307 | 13 | 48 | 271 |
| CP-19 | 484 | 349 | 16 | 135 | 271 |
| CP-20 | 391 | 390 | 1 | 1 | 387 |
| CP-21 | 247 | 241 | 2 | 6 | 234 |
| CP-22 | 610 | 465 | 15 | 145 | 351 |
| CP-23 | 235 | 170 | 3 | 65 | 127 |
| CP-26 | 389 | 372 | 2 | 17 | 354 |
| CP-27 | 130 | 130 | 0 | 0 | 129 |
| CP-28 | 238 | 237 | 0 | 1 | 234 |
| CP-30 | 523 | 507 | 3 | 16 | 495 |
| CP-32 | 527 | 246 | 59 | 281 | 86 |
| CP-33 | 449 | 396 | 40 | 53 | 354 |
| CP-34 | 462 | 445 | 7 | 17 | 431 |
| CP-36 | 386 | 260 | 108 | 126 | 150 |
| CP-37 | 342 | 221 | 43 | 121 | 142 |
| CP-38 | 406 | 306 | 29 | 100 | 234 |
| CP-39 | 518 | 513 | 2 | 5 | 506 |
| CP-40 | 509 | 126 | 101 | 383 | 34 |
| CP-41 | 349 | 332 | 5 | 17 | 313 |
| CP-42 | 346 | 244 | 10 | 102 | 171 |
| CP-43 | 460 | 343 | 90 | 117 | 249 |
| CP-44 | 321 | 319 | 1 | 2 | 315 |
| CP-45 | 357 | 213 | 111 | 144 | 115 |
| CP-47 | 537 | 511 | 12 | 26 | 488 |
| CP-48 | 637 | 522 | 7 | 115 | 418 |
| CP-49 | 451 | 434 | 19 | 17 | 416 |
| CP-52 | 728 | 461 | 47 | 267 | 288 |
| CP-53 | 562 | 561 | 0 | 1 | 558 |
| CP-55 | 793 | 373 | 102 | 420 | 171 |
| CP-56 | 742 | 574 | 9 | 168 | 444 |
| CP-57 | 507 | 506 | 0 | 1 | 503 |
| CP-58 | 525 | 523 | 1 | 2 | 519 |
| CP-59 | 405 | 405 | 0 | 0 | 404 |
| CP-60 | 512 | 503 | 0 | 9 | 494 |
| CP-61 | 397 | 396 | 0 | 1 | 393 |
| CP-63 | 610 | 608 | 1 | 2 | 604 |
| CP-64 | 382 | 377 | 8 | 5 | 370 |
| CP-65 | 369 | 364 | 1 | 5 | 357 |
| CP-66 | 468 | 467 | 1 | 1 | 464 |
| CP-68 | 327 | 198 | 30 | 129 | 116 |
| CP-69 | 587 | 585 | 0 | 2 | 581 |
| CP-70 | 422 | 308 | 55 | 114 | 239 |
| UP-01 | 239 | 96 | 72 | 143 | 42 |
| UP-04 | 258 | 249 | 2 | 9 | 241 |
| UP-06 | 458 | 338 | 36 | 120 | 266 |
| UP-07 | 376 | 370 | 4 | 6 | 364 |
| UP-08 | 257 | 256 | 0 | 1 | 253 |
| UP-09 | 286 | 267 | 5 | 19 | 257 |
| UP-10 | 165 | 136 | 9 | 29 | 108 |
| UP-11 | 417 | 392 | 2 | 25 | 370 |
| UP-12 | 339 | 186 | 27 | 153 | 121 |
| UP-13 | 106 | 95 | 2 | 11 | 86 |
| UP-14 | 340 | 322 | 9 | 18 | 302 |
| UP-15 | 214 | 212 | 1 | 2 | 208 |
| UP-16 | 221 | 187 | 24 | 34 | 160 |
| UP-17 | 613 | 598 | 6 | 15 | 591 |
| UP-18 | 350 | 348 | 1 | 2 | 344 |
| UP-20 | 617 | 609 | 2 | 8 | 599 |
| UP-21 | 305 | 302 | 0 | 3 | 297 |
| UP-23 | 565 | 562 | 4 | 3 | 557 |
| UP-24 | 349 | 327 | 12 | 22 | 307 |
| UP-27 | 269 | 234 | 33 | 35 | 198 |
| UP-29 | 146 | 145 | 1 | 1 | 142 |
| UP-30 | 228 | 228 | 12 | 0 | 226 |
| **Total** | **33265** | **28342** | **1455** | **4923** | **25359** |

**Table S5.** Number of heartbeats detected in the ECG and GCG signals of 95 VHD patients from dataset #3, along with the number of FP, FN identified in GCG signals and the number of compared IBIs.

| **Patient**  **ID #** | **R-peaks** | **TP** | **FP** | **FN** | **IBI** |
| --- | --- | --- | --- | --- | --- |
| CP-01 | 448 | 447 | 1 | 1 | 444 |
| CP-02 | 651 | 633 | 12 | 18 | 615 |
| CP-03 | 481 | 466 | 12 | 15 | 454 |
| CP-04 | 661 | 588 | 39 | 73 | 539 |
| CP-05 | 509 | 489 | 13 | 20 | 476 |
| CP-06 | 294 | 274 | 20 | 20 | 255 |
| CP-07 | 451 | 446 | 1 | 5 | 439 |
| CP-08 | 544 | 540 | 4 | 4 | 535 |
| CP-09 | 364 | 244 | 37 | 120 | 173 |
| CP-10 | 506 | 427 | 43 | 79 | 351 |
| CP-11 | 656 | 575 | 28 | 81 | 509 |
| CP-12 | 423 | 414 | 15 | 9 | 403 |
| CP-13 | 837 | 826 | 2 | 11 | 817 |
| CP-14 | 472 | 268 | 66 | 204 | 139 |
| CP-15 | 630 | 429 | 48 | 201 | 372 |
| CP-16 | 355 | 349 | 4 | 6 | 341 |
| CP-17 | 362 | 328 | 12 | 34 | 301 |
| CP-18 | 601 | 598 | 1 | 3 | 593 |
| CP-19 | 484 | 389 | 31 | 95 | 334 |
| CP-20 | 391 | 390 | 0 | 1 | 387 |
| CP-21 | 247 | 246 | 1 | 1 | 243 |
| CP-22 | 610 | 435 | 20 | 175 | 315 |
| CP-23 | 235 | 234 | 0 | 1 | 231 |
| CP-25 | 602 | 320 | 61 | 282 | 140 |
| CP-26 | 389 | 369 | 8 | 20 | 347 |
| CP-27 | 130 | 129 | 1 | 1 | 126 |
| CP-28 | 238 | 238 | 0 | 0 | 237 |
| CP-29 | 290 | 146 | 35 | 144 | 76 |
| CP-30 | 523 | 508 | 3 | 15 | 497 |
| CP-32 | 527 | 341 | 109 | 186 | 245 |
| CP-33 | 449 | 438 | 14 | 11 | 425 |
| CP-34 | 462 | 441 | 3 | 21 | 421 |
| CP-35 | 484 | 361 | 66 | 123 | 273 |
| CP-36 | 386 | 243 | 48 | 143 | 134 |
| CP-37 | 342 | 335 | 0 | 7 | 328 |
| CP-38 | 406 | 345 | 16 | 61 | 292 |
| CP-39 | 518 | 502 | 11 | 16 | 489 |
| CP-40 | 509 | 364 | 12 | 145 | 296 |
| CP-41 | 349 | 348 | 1 | 1 | 345 |
| CP-42 | 346 | 328 | 6 | 18 | 311 |
| CP-43 | 460 | 237 | 39 | 223 | 131 |
| CP-44 | 321 | 305 | 15 | 16 | 287 |
| CP-45 | 357 | 323 | 14 | 34 | 302 |
| CP-46 | 499 | 367 | 21 | 132 | 291 |
| CP-47 | 537 | 355 | 6 | 182 | 233 |
| CP-48 | 637 | 231 | 152 | 406 | 93 |
| CP-49 | 451 | 446 | 1 | 5 | 439 |
| CP-50 | 781 | 641 | 27 | 140 | 552 |
| CP-51 | 621 | 618 | 2 | 3 | 614 |
| CP-52 | 728 | 451 | 22 | 277 | 245 |
| CP-53 | 562 | 559 | 0 | 3 | 556 |
| CP-54 | 397 | 252 | 10 | 145 | 142 |
| CP-56 | 742 | 621 | 11 | 121 | 511 |
| CP-57 | 507 | 506 | 1 | 1 | 503 |
| CP-58 | 525 | 524 | 0 | 1 | 521 |
| CP-59 | 405 | 404 | 0 | 1 | 401 |
| CP-60 | 512 | 492 | 7 | 20 | 481 |
| CP-61 | 397 | 397 | 0 | 0 | 396 |
| CP-62 | 572 | 498 | 1 | 74 | 434 |
| CP-63 | 610 | 609 | 0 | 1 | 606 |
| CP-64 | 382 | 381 | 6 | 1 | 378 |
| CP-65 | 369 | 369 | 0 | 0 | 368 |
| CP-66 | 468 | 422 | 0 | 46 | 384 |
| CP-67 | 539 | 476 | 28 | 63 | 425 |
| CP-68 | 327 | 313 | 19 | 14 | 299 |
| CP-69 | 587 | 584 | 1 | 3 | 579 |
| CP-70 | 422 | 354 | 25 | 68 | 315 |
| UP-01 | 239 | 146 | 32 | 93 | 102 |
| UP-02 | 286 | 208 | 55 | 78 | 154 |
| UP-03 | 264 | 125 | 42 | 139 | 75 |
| UP-04 | 258 | 225 | 11 | 33 | 198 |
| UP-05 | 462 | 331 | 38 | 131 | 241 |
| UP-06 | 458 | 174 | 76 | 284 | 65 |
| UP-07 | 376 | 372 | 1 | 4 | 366 |
| UP-08 | 257 | 255 | 2 | 2 | 251 |
| UP-09 | 286 | 268 | 12 | 18 | 255 |
| UP-10 | 165 | 155 | 9 | 10 | 144 |
| UP-11 | 417 | 332 | 21 | 85 | 263 |
| UP-12 | 339 | 296 | 27 | 43 | 269 |
| UP-13 | 106 | 96 | 6 | 10 | 87 |
| UP-14 | 340 | 340 | 1 | 0 | 338 |
| UP-15 | 214 | 214 | 0 | 0 | 213 |
| UP-16 | 221 | 167 | 38 | 54 | 126 |
| UP-17 | 613 | 539 | 18 | 74 | 493 |
| UP-18 | 350 | 348 | 2 | 2 | 344 |
| UP-19 | 116 | 67 | 40 | 49 | 37 |
| UP-20 | 617 | 328 | 23 | 289 | 193 |
| UP-21 | 305 | 304 | 0 | 1 | 301 |
| UP-23 | 565 | 565 | 4 | 0 | 563 |
| UP-24 | 349 | 326 | 9 | 23 | 309 |
| UP-25 | 244 | 163 | 44 | 81 | 127 |
| UP-26 | 160 | 157 | 1 | 3 | 153 |
| UP-27 | 269 | 156 | 98 | 113 | 74 |
| UP-29 | 146 | 144 | 1 | 2 | 140 |
| UP-30 | 228 | 189 | 41 | 39 | 150 |
| **Total** | **40527** | **34516** | **1864** | **6011** | **30765** |

**Table S6.** Number of heartbeats detected on the 6 dHF-FCG and ECG signals presented in dataset #4, along with the number of FP, FN identified in dHF-FCG signals and the number of compared IBIs.

| **Patient**  **ID #** | **R-peaks** | **TP** | **FP** | **FN** | **IBI** |
| --- | --- | --- | --- | --- | --- |
| 1 | 378 | 378 | 0 | 0 | 377 |
| 2 | 192 | 189 | 2 | 3 | 185 |
| 3 | 166 | 165 | 0 | 1 | 164 |
| 4 | 205 | 198 | 7 | 7 | 190 |
| 5 | 337 | 337 | 0 | 0 | 336 |
| 6 | 156 | 155 | 1 | 1 | 153 |
| **Total** | **1434** | **1422** | **10** | **12** | **1405** |
